# Prospective study evaluating dynamic changes of cell-free HPV DNA in locoregional viral-associated oropharyngeal cancer treated with induction chemotherapy and response-adaptive treatment

**DOI:** 10.1186/s12885-021-09146-z

**Published:** 2022-01-03

**Authors:** Ari J. Rosenberg, Evgeny Izumchenko, Alexander Pearson, Zhen Gooi, Elizabeth Blair, Theodore Karrison, Aditya Juloori, Daniel Ginat, Nicole Cipriani, Mark Lingen, Hillary Sloane, Daniel L. Edelstein, Kirsten Keyser, Johannes Fredebohm, Frank Holtrup, Frederick S. Jones, Daniel Haraf, Nishant Agrawal, Everett E. Vokes

**Affiliations:** 1 Department of Medicine, Section of Hematology and Oncology, University of Chicago, Chicago, IL USA; 2 Section of Otolaryngology-Head and Neck Surgery, University of Chicago, Chicago, IL USA; 3 Department of Public Health Sciences, University of Chicago, Chicago, IL USA; 4 Department of Radiation and Cellular Oncology, University of Chicago, Chicago, IL USA; 5 Department of Radiology, University of Chicago, Chicago, IL USA; 6 Department of Pathology, University of Chicago, Chicago, IL USA; 7 Sysmex Inostics, Inc., Baltimore, MD USA; 8 Sysmex Inostics GmbH, Hamburg, Germany

**Keywords:** Head and neck cancer, Human papillomavirus, Chemotherapy, Radiotherapy, Treatment de-escalation

## Abstract

**Background:**

Human papillomavirus (HPV)-associated oropharyngeal cancer (OPC) has a favorable prognosis which has led to efforts to de-intensify treatment. Response-adaptive de-escalated treatment is promising, however improved biomarkers are needed. Quantitative cell-free HPV-DNA (cfHPV-DNA) in plasma represents an attractive non-invasive biomarker for grading treatment response and post-treatment surveillance. This prospective study evaluates dynamic changes in cfHPV-DNA during induction therapy, definitive (chemo)radiotherapy, and post-treatment surveillance in the context of risk and response-adaptive treatment for HPV + OPC.

**Methods:**

Patients with locoregional HPV + OPC are stratified into two cohorts: High risk (HR) (T4, N3, $\ge$ 20 pack-year smoking history (PYH), or non-HPV16 subtype); Low risk (LR) (all other patients). All patients receive induction chemotherapy with three cycles of carboplatin and paclitaxel. LR with ≥ 50% response receive treatment on the single-modality arm (minimally-invasive surgery or radiation alone to 50 Gy). HR with ≥ 50% response or LR with ≥ 30% and < 50% response receive treatment on the intermediate de-escalation arm (chemoradiation to 50 Gy with cisplatin). All other patients receive treatment on the regular dose arm with chemoradiation to 70 Gy with concurrent cisplatin. Plasma cfHPV-DNA is assessed during induction, (chemo)radiation, and post-treatment surveillance. The primary endpoint is correlation of quantitative cfHPV-DNA with radiographic response.

**Discussion:**

A de-escalation treatment paradigm that reduces toxicity without compromising survival outcomes is urgently needed for HPV + OPC. Response to induction chemotherapy is predictive and prognostic and can select candidates for de-escalated definitive therapy. Assessment of quantitative cfHPV-DNA in the context of response-adaptive treatment of represents a promising reliable and convenient biomarker-driven strategy to guide personalized treatment in HPV + OPC.

**Trial registration:**

This trial is registered with ClinicalTrials.gov on October 1^st^, 2020 with Identifier: NCT04572100.

## Background

Over the past several decades, there has been a dramatic increase in incidence of Human Papillomavirus (HPV)-associated Oropharyngeal Cancer (OPC) despite a reduction in smoking-related head and neck cancer[1]. A causal association between high-risk HPV subtypes and OPC has been established, with HPV-16 being the most commonly implicated subtype[1]. Oncogenic HPV leads to malignant transformation through integration of the viral genome elements into host genome and/or episomal state [2]. The expression of HPV-16 specific E7 and E6 oncogenic proteins leads to downregulated pRb and p53 and upregulated p16, mechanistically driving viability of oropharyngeal cancer cells[3]. Clinical trials evaluating combined modality therapy with chemotherapy and radiation treatment have demonstrated a favorable prognosis for HPV + OPC compared with HPV-negative disease, with 3-year overall survival (OS) rates of approximately 80–90%[4–6].

Current treatment paradigms for locoregionally advanced HPV + OPC include definitive concomitant chemoradiation or surgical resection followed by adjuvant radiation with or without chemotherapy [7]. However, standard combined modality therapy is associated with substantial acute and long-term toxicities. This has led to interest in developing a de-intensification treatment paradigm for HPV + OPC that optimizes the therapeutic to toxicity ratio for patients [8, 9]. Strategies to de-escalate treatment for patients with HPV + OPC have included replacing or omitting concurrent chemotherapy [10–12], dose or volume reduction of concurrent chemoradiation[13, 14], de-intensified adjuvant therapy[15, 16], and response adaptive de-intensification[9, 17–19]. Attempts to de-intensify treatment in randomized phase III trials by reducing or omitting chemotherapy in HPV + OPC resulted in worse survival[10, 11], suggesting that the favorable prognosis is related to the increased sensitivity of HPV + OPC to chemotherapy and radiation therapy [20].

Response to induction chemotherapy represents an attractive strategy to select candidates for treatment de-escalation in HPV + OPC. The rationale for this strategy is based on the observation that a favorable response to induction therapy is associated with superior prognosis following subsequent definitive chemoradiation[19, 21]. Distant recurrence in HPV-associated OPC is as high as 15% in some studies[22], suggesting a role for intensified systemic therapy. Radiotherapy volume and dose in the context of concurrent chemoradiotherapy is a substantial driver of acute and long-term toxicity of this therapeutic approach. Risk and response adaptive de-escalated chemoradiation is a promising strategy to dynamically select patients for de-intensified definitive treatment[19, 20]. The OPTIMA trial[19] demonstrated that risk and response stratified locoregional treatment resulted in excellent outcomes with 2-year OS of 100% and 97% for low-risk and high-risk cohorts respectively, and acute toxicities were significantly reduced in the de-escalated cohorts[19].

Improved strategies to monitor and adapt treatment response to personalize patient de-intensification is urgently needed. The use of reliable quantitative blood-based biomarkers represents an appealing approach to dynamically monitor treatment response during induction therapy and definitive treatment, as well as following completion of definitive therapy to monitor for disease recurrence. It has been shown that cell free HPV-DNA (cfHPV-DNA) can be detected in plasma of patients with HPV + OPC. A number of retrospective studies have reported that circulating tumor DNA from high-risk HPV subtypes is detectable in the plasma of patients with HPV + OPC using real-time qPCR and droplet digital PCR (ddPCR) [23–31]. These results suggest that cfHPV-DNA in plasma may predict disease recurrence prior to radiography[32]. However, despite increasing interest in noninvasive HPV detection and promising preliminary data, there is no routine plasma-based testing method for patients with HPV associated disease.

**Fig. 1:**
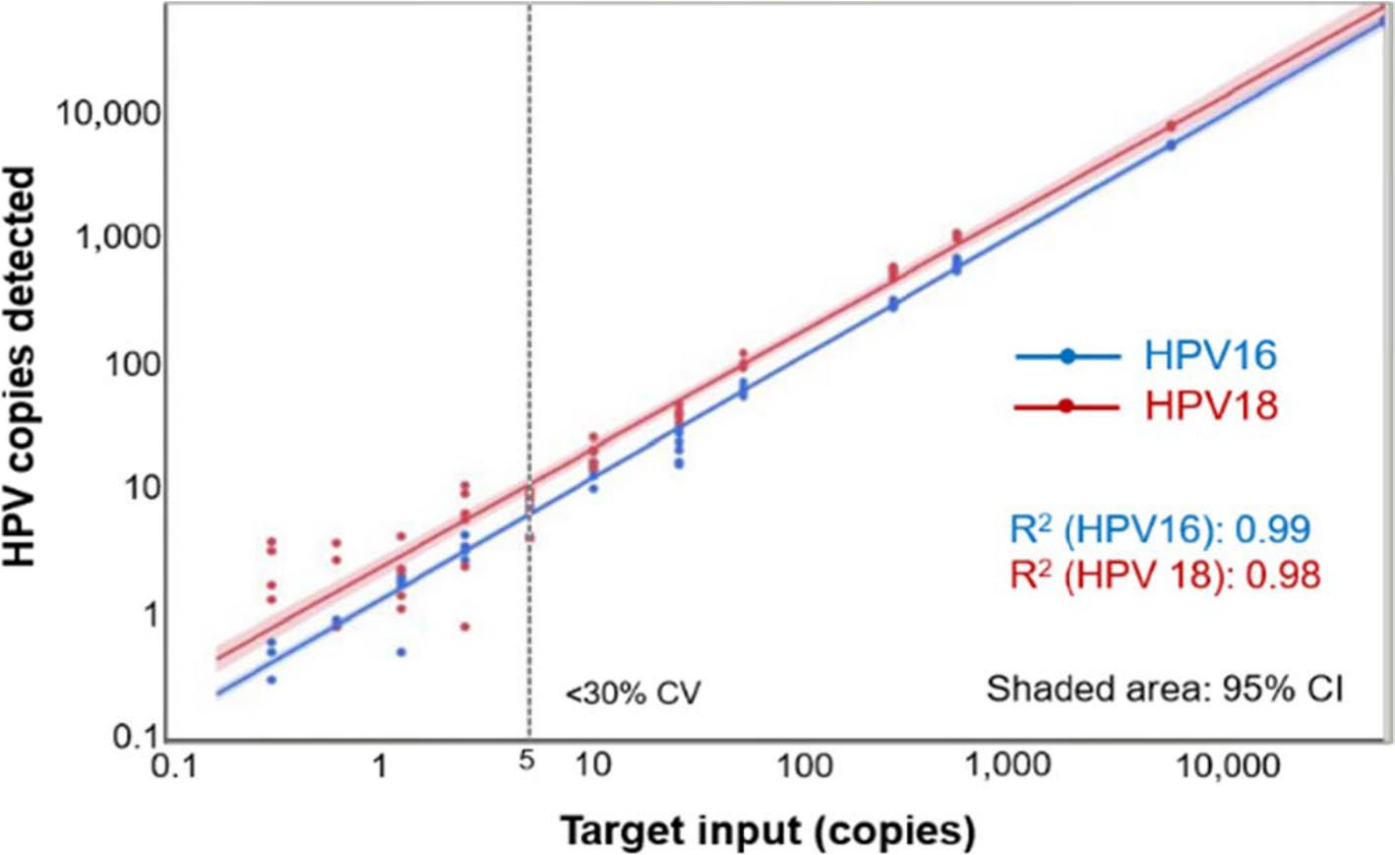
Quantification of HPV 16 and 18 in contrived samples. Dilution series of samples tested in replicate at 12 tiers ranging from 0.3 to 50,000 copies

The HPV-SEQ test is an NGS-based method based on Safe-SeqS technology[33] that enables highly sensitive detection and quantification of HPV16/18 DNA in the plasma of OPC patients[34]. Analytical performance characterization studies revealed robust quantitative detection of HPV 16/18 DNA across a dynamic range over 5 orders of magnitude (Fig. 1), and a low level of background signal (< 0.04 copies per sample across 20 healthy donors), indicating high analytical sensitivity and specificity. The incorporation of this novel approach in the context of our institutional treatment de-escalation paradigm for the treatment of locoregional HPV + OPC as a dynamic biomarker of treatment response and surveillance is a logical next step in clinical and translational application.

The present study investigates the utility of serial cfHPV-DNA assessments in a cohort of locoregional HPV + OPC patients treated with induction chemotherapy followed by risk and response adaptive de-intensified treatment.

## Methods/Design

This study protocol was approved by the University of Chicago Institutional Review Board (UCCCC IRB Number 20–0713). All patients provide written informed consent prior to enrollment. The study is funded by the American Cancer Society Institutional Research Grant (IRG-19–136-59).

### Study design

**Fig. 2:**
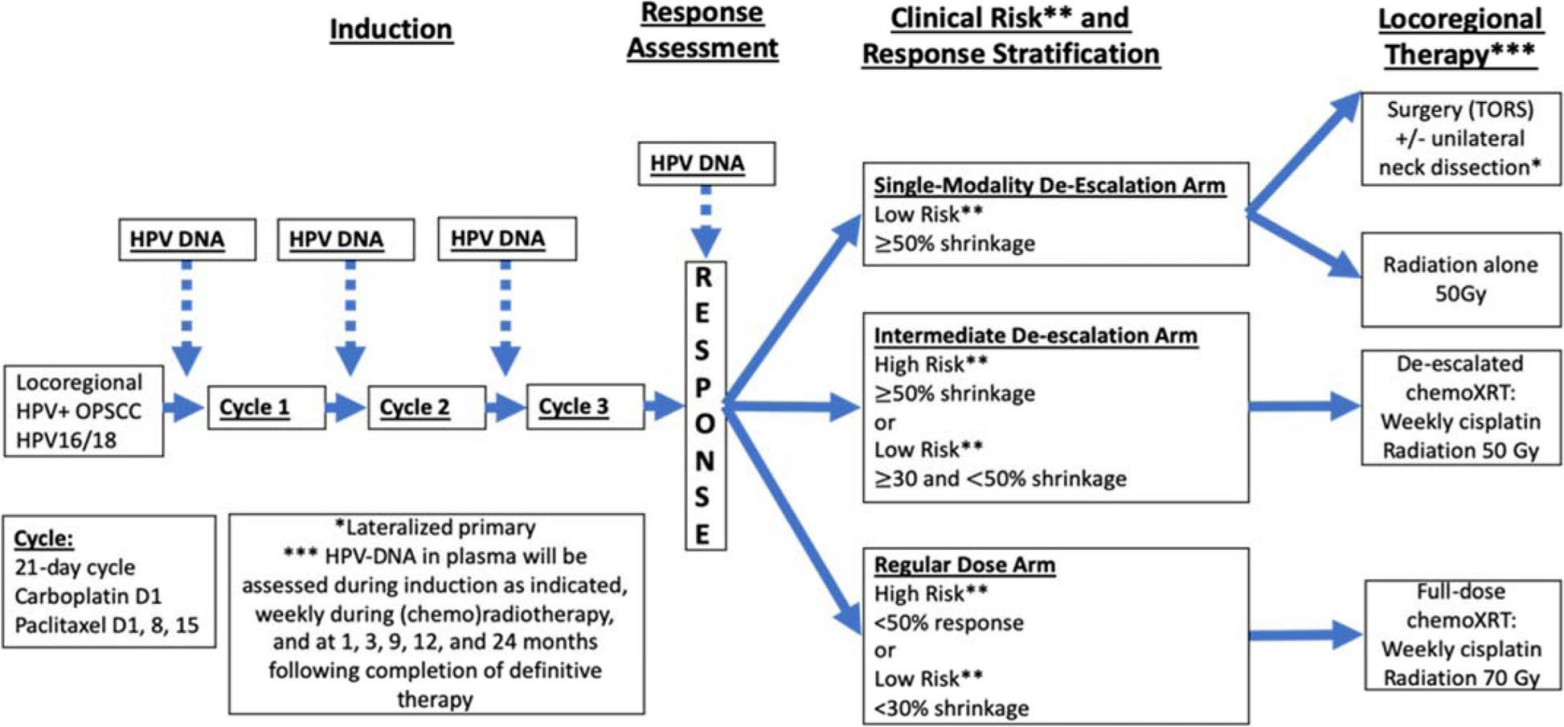
Clinical trial schema for “Prospective Study evaluating dynamic changes of HPV DNA in locoregional viral-associated oropharyngeal cancer treated with induction chemotherapy followed by risk and response-adaptive treatment.” Patients with locoregional HPV16 or HPV18 OPSCC receive 3 cycles of induction carboplatin and paclitaxel followed by risk and response-based adaptive de-escalated treatment with single modality (TORS or radiation alone to 50 Gy), intermediate de-escalation (chemoradiation to 50 Gy with weekly cisplatin), or regular dose (chemoradiation to 70 Gy with weekly cisplatin). All patients receive quantitative HPV-DNA of plasma during each cycle of induction, weekly during radiation-based treatment, and at 1, 3, 6, 12, 18, and 24 months following completion of definitive treatment

The study is designed as a single-arm, single-center, prospective study with co-primary endpoints to assess the feasibility of serial quantitative cfHPV-DNA analysis and to assess correlation with radiographic response in HPV + OPC patients undergoing induction chemotherapy followed by risk and response-stratified de-escalated therapy at the University of Chicago. Secondary endpoints include: 1) assessing changes in cfHPV-DNA during response-stratified (chemo)radiotherapy, 2) assessing the feasibility of cfHPV-DNA testing for surveillance following completion of definitive treatment, 3) assessing feasibility and toxicity of weekly cisplatin-based de-escalated chemoradiation, 4) estimating the pathologic response in patients undergoing TORS following induction chemotherapy, and 5) evaluating progression-free survival (PFS), overall survival, locoregional control (LRC), and distant control (DC). Exploratory endpoints include late-toxicities including enteral tube dependence, and quality of life in patients receiving de-escalated (chemo)radiation. Accrual began in November 2020 and the study will continue to accrue. The study schema is presented in Fig. 2.

### Subjects

Adult patients with locoregionally advanced HPV-associated OPC are eligible. Key inclusion and exclusion criteria are presented in Table 1.

**Table 1:** Key inclusion and exclusion criteria

Key inclusion criteria	Key exclusion criteria
Patients must be at least 18 years of age	Unequivocal demonstration of distant metastatic disease
Pathologically confirmed HPV + OPC^1^	Non-HPV16/18 subtype
Subjects with AJCC (8^th^ edition, 2018) N1 (if single lymph node must be $\ge$ 3 cm), N2-N3 nodal disease or T3-T4 primary tumor	N2-3 (or nodal conglomerate $\ge$ 6 cm)
Measurable disease by RECIST 1.1 criteria	> 20 pack year smoking history
No previous radiation or chemotherapy for head and neck cancer	HPV18 subtype
No complete surgical resection for head and neck cancer	Unidentifiable primary site
ECOG performance status 0–1	Intercurrent medical illness which would impair patient tolerance to therapy or limit survival
Normal organ function	History of HIV, active hepatitis B or hepatitis C

^1^Defined by p16 positivity by immunohistochemistry with confirmation with HPV PCR confirming HPV subtype

### Assessments

Prior to treatment, all patients will undergo physical examination, pan-endoscopy with biopsy, baseline CT or MRI of the head and neck and CT chest, PET/CT scan as recommended, multidisciplinary team recommendation, and baseline laboratory assessments. All patients will have completed dental evaluation and speech and swallowing consultation before or during induction therapy. Prior to initiation of treatment, all patients will be categorized as either high-risk or low risk per the criteria noted in Table 2.

**Table 2:** Pre-treatment Clinical Risk Assessment

Clinical Risk Assessment (based on pre-treatment assessment)	
Low Risk (All of the below)	High Risk (Any of the below)
T0-T3	T4
N0-N1	N2-3 (or nodal conglomerate $\ge$ 6 cm)
$\le$ 20 pack year smoking history	> 20 pack year smoking history
HPV16 subtype	HPV18 subtype

All patients will be monitored with physical examination and laboratory assessments weekly during induction chemotherapy including plasma HPV-DNA collection as indicated in Fig. 2. Repeat imaging of the head and neck will be performed with CT or MRI within 10 days of cycle 3 day 15 of induction therapy for response-stratification of locoregional therapy. Additional follow-up imaging of the head and neck with CT or MRI and PET/CT will be performed at 12 weeks following completion of definitive therapy.

Quality of life will be assessed in all patients at 1 year following completion of TORS or (chemo)radiation. These quality of life assessments will include Performance Status Scale for Head and Neck Cancer Patients (PSS-HN), Functional Assessment of Cancer Therapy – Head and Neck Version 4 (FACT – H&N), and MD Anderson – Dysphagia Index.

## Treatment

### Induction chemotherapy

Induction chemotherapy will be administered on an outpatient basis and is detailed in Fig. 2. Carboplatin and paclitaxel combination will be administered for three cycles of three weeks duration each with selected dose delays and modifications as outlined in Table 3. Paclitaxel is administered at 100 mg/m^2^ on days 1, 8, and 15, and carboplatin is administered at AUC 5 on day 1 with a baseline creatinine level drawn within 1 week prior to starting chemotherapy. Anti-emetic support, steroids, hydration, and figrastim is administered per institutional standards.

**Table 3:** Selected dose modifications during induction therapy

Adverse Reaction	Occurrence	Paclitaxel Dose (mg/m^2^)	Carboplatin Dose (AUC mg·min/mL)
**Hematologic**			
ANC < 1500/mm^3^ OR ANC < 500/mm^3^ for more than 7 days	First	75	4.5
	Second	50	3
	Third	Discontinue Treatment	
Platelet count less than 100,000/mm^3^	First	75	4.5
	Second	Discontinue Treatment	
**Neurotoxicity (Peripheral)**			
Grade 2	First	75	
	Second	50	
	Third	Discontinue Treatment	
Grade 3–4	First	Withhold paclitaxel, until improves to < = grade 1, then resume at one lower dose level	

### Risk and Response Stratified Grouping

Patients will be assigned to a) single-modality de-escalation arm (SDA), b) intermediate de-escalation arm (IDA), or c) regular-dose arm (RDA) based on risk and response stratification. SDA includes patients who are low-risk (Table 2*)*, and have $\ge$ 50% tumor shrinkage by RECIST. These patients are treated with either TORS (T1-T2 with primary $\le$ 3 cm), or RT alone to 50 Gy.

Patients assigned to the IDA will include low-risk disease with < 50% but ≥ 30% reduction of tumor or patients who have high-risk disease and ≥ 50% reduction of tumor. These patients will be treated with de-intensified CRT to 50 Gy with concurrent cisplatin 40 mg/m2 weekly for 5 doses.

Patients who have low-risk disease and < 30% reduction of tumor or patients who have high-risk disease and < 50% reduction of tumor will receive treatment on the RDA with CRT to 70 Gy with concurrent cisplatin 40 mg/m2 weekly for 7 doses.

The weekly cisplatin-based concurrent chemoradiation platform was chosen based on the potential for weekly adaptive de-escalation in future paradigms built on data from this prospective study. This platform is currently widely utilized and familiar across institutions and therefore has the potential to be highly adaptable and broadly applicable.

### Response-adaptive volume de-escalation

Patients who have $\ge$ 50% tumor shrinkage by RECIST criteria will also receive radiation-adaptive volume de-escalation[19, 20]. Patients treated on the SDA with RT alone to 50 Gy will be treated with 50 Gy to gross tumor volume with margin but no elective nodal RT. Patients on the IDA will receive RT with concurrent cisplatin as described above with 50 Gy to gross tumor volume with a margin but no elective nodal RT. Patients treated on the regular dose arm who have $<$ 50% tumor shrinkage per RECIST will receive 70 Gy to gross tumor volume and 50 Gy to elective nodal volume.

### Adjuvant radiation post-operatively

Select patients that undergo TORS may have indication to receive adjuvant radiation. In the absence of adverse pathologic features, patients will not receive adjuvant radiation therapy following induction chemotherapy and TORS. However, post-operative radiation therapy will be administered for adverse pathologic features. For perineural or lymphovascular invasion, radiation to 40 Gy in 2 Gy once daily fractions will be administered. For extracapsular extension of positive surgical margins, radiation to 44 Gy in 2 Gy once daily fractions will be administered. The post-operative radiation volumes are at the discretion of the treating physician but generally will be targeted toward the surgical bed site with adverse pathology and should begin within 4 weeks and no later than 6 weeks after surgical resection.

## Statistical design

For this prospective study, 36 patients are anticipated to enroll and receive treatment. The co-primary endpoints of this study are to assess the feasibility of measuring serial quantitative cfHPV-DNA in patients undergoing induction chemotherapy followed by risk and response-stratified de-escalated therapy for HPV-associated OPC, and to assess correlation of serial plasma cfHPV-DNA levels with radiographic response to induction therapy. The approach will be considered feasible if at least 85% of patients (*n* = 31) complete induction therapy with quantitative cfHPV-DNA evaluation performed at all four planned time-points. Logistic regression will be performed to determine the association between cfHPV-DNA levels and radiographic response.

The secondary endpoints of this study include assessing changes in plasma cfHPV-DNA during response-stratified (chemo)radiotherapy, evaluating cfHPV-DNA for surveillance following completion of definitive treatment, correlating plasma cfHPV-DNA changes with sputum HPV-DNA, assessing feasibility and toxicity of weekly cisplatin-based de-escalated chemoradiation, estimating the pathologic response in patients undergoing TORS following induction chemotherapy, and evaluating progression-free survival, overall survival, locoregional control, and distant control. Changes in plasma cfHPV-DNA levels will be analyzed by fitting mixed effects models for longitudinal data. Pearson correlation coefficients will be computed to indicate the correlation between plasma and saliva HPV-DNA levels. If appropriate, Bland–Altman plots will also be constructed. Adverse events will be summarized by type, grade, and attribution. The pathologic complete response in patients who receive TORS will be reported along with an exact 90% confidence interval. PFS and OS, stratified by risk group, will be depicted using Kaplan–Meier (1958) plots. Locoregional and distant control rates will be assessed descriptively.

Exploratory objectives include late-toxicities including enteral tube dependence, and quality of life in patients receiving de-escalated (chemo)radiation. Acute and late toxicity, including the degree of dysphagia at 1 year, will be summarized by type, grade, and attribution. Quality of life measures will be analyzed using mixed effects regression modeling to assess trends over time.

## Discussion

Over the past several decades, the incidence of HPV-related OPC has been increasing at an alarming rate and this trend is anticipated to continue[35, 36]. The survival of HPV-related OPC is significantly improved compared with HPV-negative head and neck cancer[6, 37]. The current treatment paradigm for locoregionally advanced disease remains a multimodality therapeutic approach of definitive chemoradiation or upfront surgical resection followed by adjuvant (chemo)radiation, and is associated with substantial treatment related morbidity[7, 38]. Active investigation to identify a de-escalation paradigm that optimizes survival while reducing treatment-related morbidity for this patient population is ongoing, however the optimal strategy remains undefined[9, 39].

A strategy to replace concurrent chemotherapy with cetuximab or to omit concurrent chemotherapy has demonstrated worse survival compared with definitive radiation with concurrent cisplatin in setting of worse locoregional control[10–12, 40]. A de-escalation paradigm evaluated in the E3311 trial of transoral surgery followed by risk-adaptive de-intensified adjuvant treatment for intermediate pathologic risk is demonstrating promising 3-year progression free and overall survival; however, this strategy has yet to be compared with a definitive chemoradiation approach[15].

An alternative approach being investigated involves adaptive treatment de-intensification based on the hypothesis that a personalized treatment intensity can be achieved by assessing tumor response to treatment. Response-adaptive de-escalation following induction therapy that uses radiographic response to select patients for treatment de-intensification has been evaluated with promising results[17–20]. The 30 ROC trial used functional imaging to assess hypoxia with ^18^F-FMISO (fluoromisonidazole) PET scan at baseline and at day 10 of concurrent chemoradiation. Patients without hypoxia at baseline or after treatment received adaptive de-escalated chemoradiation to 30 Gy with concurrent chemotherapy[14]. However, these de-escalation trials enroll exclusively low risk HPV + patients, hence a personalized strategy for an inclusive HPV + cohort remains of great interest.

The incorporation of a reliable blood-based biomarker to guide adaptive de-escalated therapy is a rational and logical next step in treatment optimization for HPV + OPC patients. There is opportunity to explore reliable quantitative blood-based biomarkers during induction, response-adaptive definitive treatment, and following completion of therapy to monitor for disease recurrence and facilitate salvage treatment. This prospective study is set to evaluate the dynamics of quantitative cfHPV-DNA levels as a reliable blood-based biomarker in combination with established response-adaptive treatment de-intensification, and provide the data needed to develop a blood-based biomarker driven strategy. Early incorporation in the context of a multimodality treatment approach for HPV + OPC has suggested that quantitative HPV-DNA dynamics seem to correlate with treatment response and may have potential to predict disease recurrence prior to radiographic or clinical evidence of disease[32].

Data from this prospective study will be invaluable in the design of a subsequent larger proof-of-concept clinical trial incorporating dynamic changes in quantitative HPV-DNA as a component of treatment response assessment, adaptive de-intensification, and monitoring for recurrence. Beyond this exciting application, the potential of NGS-based quantitative HPV-DNA analysis in the context of clinical trial research is broad. Evaluating the HPV integration status (integrated versus episomal) and identification of specific gene mutations in high-risk HPV OPC can also be explored. Furthermore, the HPV-SEQ assay allows parallel interrogation of genes commonly mutated in head and neck malignancies. Such multi-functional assays to synergize somatic drivers and HPV detection in patients’ plasma may further facilitate personalized treatment decisions incorporating tumor biology. Finally, there is additional opportunity to incorporate this assay for treatment optimization in patients with other HPV-associated malignancies such as cervical and anal cancer.

## Conclusion

This prospective study evaluating dynamic changes of HPV DNA levels in locoregional viral-associated oropharyngeal cancer treated with induction chemotherapy followed by risk and response-adaptive treatment will provide initial data to develop a personalized, adaptive, de-escalation strategy in HPV-associated OPC.

## Data Availability

Data sharing is not applicable to this article as no datasets have thus far been generated or analyzed, but will be available at the time of completion and analysis.

## Abbreviations

HPV: Human papillomavirus
OPC: Oropharyngeal cancer
OS: Overall survival
cfHPV-DNA: Cell free HPV DNA
ddPCR: Droplet digital PCR
PFS: Progression free survival
LRC: Locoregional control
DC: Distant control
PSS-HN: Performance Status Scale for Head and Neck Cancer Patients
FACT – H&N: Functional Assessment of Cancer Therapy – Head and Neck Version 4
SDA: Single-modality de-escalation arm
IDA: Intermediate de-escalation arm
RDA: Regular-dose arm
TORS: Transoral robotic surgery
18F-FMISO: Fluoromisonidazole
